# Thrombotic Microangiopathy After Lung Transplantation: A Retrospective Observational Multicenter Cohort Study

**DOI:** 10.1016/j.jhlto.2025.100335

**Published:** 2025-07-08

**Authors:** Pierre Gazengel, Vincent Bunel, Kinan El-Husseini, Mohamad Zaidan, Edouard Lefevre, Romain Kessler, Xavier Demant, Loïc Falque, Emmanuel Eschapasse, Thomas Villeneuve, Gaelle Dauriat, Pauline Pradère, Olaf Mercier, Elie Fadel, Clément Picard, Jérôme Le Pavec

**Affiliations:** a Pulmonology and Lung Transplantation Department, Marie-Lannelongue Hospital, Groupe Hospitalier Paris-Saint-Joseph, Le Plessis-Robinson, France; b Paris–Sud University, School of Medicine, Paris-Saclay University, Le Kremlin Bicêtre, France; c INSERM, Research Unit UMR_S 999, Paris-Sud University, Marie-Lannelongue Hospital, Groupe Hospitalier Paris-Saint-Joseph, Le Plessis Robinson, France; d Pulmonology Department, Bichat University Hospital, AP-HP, Paris, France; e Nephrology Department, Kremlin-Bicêtre University Hospital, AP-HP, Le Kremlin-Bicêtre, France; f Pulmonology Department, Lung Transplantation Group, Strasbourg University Hospitals, Strasbourg, France; g Respiratory Medicine Department, Haut-Lévêque Hospital, Bordeaux University, Pessac, France; h Pulmonology and Physiology Department, Grenoble-Alpes University Hospital, Thorax and Vessels Hub, Grenoble, France; i Pulmonology Department, Nord Laennec Hospital, Nantes University Hospital, Nantes, France; j Pulmonology Department, Toulouse University Hospital, Toulouse, France; k Thoracic, Vascular, and Heart-Lung Transplantation Surgery, Service de Chirurgie Thoracique, Vasculaire et Transplantation Cardio-pulmonaire, Marie-Lannelongue Hospital, Groupe Hospitalier Paris-Saint-Joseph, Le Plessis Robinson, France; l Pulmonology Department, Foch Hospital, Suresnes, France

**Keywords:** Lung transplantation, Thrombotic microangiopathy, Belatacept, Renal function, Infection

## Abstract

**Background:**

Thrombotic microangiopathy (TMA) is a well-recognized complication of solid-organ transplantation that chiefly affects the kidneys. The objective of this study was to describe TMA features and outcomes after lung transplantation.

**Patients and methods:**

This retrospective observational study included patients with TMA following lung or heart-lung transplantation at eight French centers in 2006–2023. Univariate and multivariate analyses were done to identify factors associated with outcomes.

**Results:**

Of the 4565 patients, 82 (1.8%) experienced TMA, at a median of 19 [6−34] months after transplantation; among them, 79 were included (51% female; median age 50 [33−61] years). Mortality during the median follow-up of 31 [11−66] months was 38/79 (48%). Etiological factors were above-target calcineurin inhibitor (CNI) trough levels (48%), combined CNI and mTOR inhibitor therapy (23%), and infection (9%). CNI was continued in 70 patients and replaced by belatacept in 9 patients. In the belatacept group, renal function at one year was better but death, bacterial pneumonia, and CMV viremia were more common; none of the differences was significant, perhaps given the small sample size.

**Conclusion:**

Mortality was high after TMA in lung or heart-lung transplant recipients. CNI monitoring protocols should be improved to minimize the risk of toxicity. Belatacept instead of CNI therapy was associated with better kidney function but also with higher frequencies of adverse events, suggesting a need for great caution. Studies adequately powered to assess the risk/benefit ratio of belatacept therapy according to the dosing regimen, patient features, and concomitant immunosuppressants are needed.

## Introduction

Thrombotic microangiopathy (TMA) is a pathologic entity in which microvascular endothelial lesions cause microangiopathic hemolytic anemia and microthrombi with consumptive thrombocytopenia.^1^ Injury to the organs supplied by the affected vascular beds develops rapidly. TMA exists as several primary and secondary diseases caused by very different mechanisms. Among causes of secondary TMA, transplantation is well recognized, although incidence data are scant.^2^ The kidneys are usually the organs most severely affected by transplantation-related TMA. TMA is an emergency, and both morbidity and mortality are high in the absence of prompt diagnosis and treatment.^3^, ^4^

The features of TMA after transplantation vary with the nature of the transplanted organ. Studies focused specifically on lung transplant (LTx) recipients are scarce.^5^, ^6^, ^7^, ^8^, ^9^, ^10^, ^11^ One of the largest cohorts, published by Hachem and colleagues, included 24 episodes in 20 patients, with a mean time from LTx to TMA of 449±422 days (range, 17 days to 4 years) and a median survival of 377 days.^5^ Whether TMA affects allograft function is unclear. In a study of 31 patients with TMA after kidney transplantation, graft survival was lower than in a propensity-matched group without TMA.^12^ Among liver-transplant recipients, lower survival has been reported in patients with vs. without TMA.^13^ Data on outcomes of LTx recipients after TMA, notably regarding graft function, are lacking.

The pathogenic mechanisms of TMA after LTx remain incompletely elucidated and may vary across patients, given the reported differences in clinical presentation and time from LTx to TMA. Risk factors for TMA include female sex,^5^ history of TMA,^5^ and non-immune partial ADAMTS 13 deficiency.^14^ Macrolide therapy may increase the risk of TMA.^6^ Among immunosuppressants used in transplant recipients, calcineurin inhibitors (CNIs) can damage the endothelium.^6^, ^7^, ^9^, ^10^ Combined treatment with a CNI and a mammalian target of rapamycin (mTOR) inhibitor was associated with a 6- to 7-fold increase in the risk of TMA compared to either drug class alone in LTx recipients.^5^ When immunosuppressant toxicity is suspected, switching to belatacept has been suggested. Belatacept belongs to a new class of immunosuppressants that inhibit T-cell co-stimulatory pathways. However, belatacept in TMA after transplantation has been studied chiefly in kidney-transplant recipients, in whom better renal function was noted.^15^, ^16^, ^17^, ^18^, ^19^ Data in LTx recipients are scant^20^, ^21^, ^22^, ^23^ Better knowledge of the features and risk factors of TMA after LTx might help to improve the effectiveness and personalization of treatment, notably regarding the potential indications of belatacept therapy.

The primary objective of this retrospective observational multicenter cohort study was to determine survival after TMA in LTx recipients in France. The secondary objectives were to assess the clinical features, renal and lung-graft outcomes, and effects of switching from CNI to belatacept therapy in LTx recipients with TMA.

## Methods

This retrospective study of de-identified data was conducted in compliance with the Declaration of Helsinki and was approved by the Paris Saint Joseph Hospital institutional review board (Groupe Éthique en Recherche Médicale, # IRB548). The data were managed in compliance with requirements by the French data protection authority (*Commission Nationale de l′Informatique et des Libertés*).

### Study design

We retrospectively reviewed the medical files of consecutive patients who underwent cadaveric heart-lung transplantation (HLTx) or LTx between January 2006 and December 2023 at any of eight French centers and who subsequently experienced TMA. In France, 2006 was marked by the introduction of a high-emergency lung-transplant allocation program and by a considerable expansion in the use of extracorporeal life support.

### Patient management and follow-up

Induction therapy and maintenance immunosuppression were given according to local protocols at each center. All patients received life-long cotrimoxazole for *Pneumocystis jirovecii* pneumonia prophylaxis. Valganciclovir for CMV prophylaxis was prescribed according to local protocols.

Patients were monitored routinely for primary graft dysfunction (PGD). Monitoring transbronchial biopsies were obtained either at set intervals or when clinically indicated, depending on the protocol at each center. The PGD grade was defined according to the International Society for Heart and Lung Transplantation, based on the ratio of arterial partial oxygen pressure over fraction of inspired oxygen and the presence of infiltrates within the allograft or allografts.^24^ Patients with allograft dysfunction were investigated for acute cellular rejection, lymphocytic bronchiolitis/neutrophilic reversible allograft dysfunction, and airway injury caused by infection/colonization. Chronic lung allograft dysfunction (CLAD) was diagnosed when the forced expiratory volume in one second and/or forced vital capacity declined to ≤80% of the best postoperative value.^25^ Both parameters were evaluated during routine outpatient assessments, starting at least three months after transplantation. Comprehensive lung-function tests including spirometry and lung-volume measurements, high-resolution computed tomography of the chest, and bronchoscopy with bronchoalveolar lavage and transbronchial biopsy were performed to look for causes of lung-allograft dysfunction such as persistent acute rejection, azithromycin-responsive allograft dysfunction, infection, anastomotic stricture, and sarcoidosis recurrence.

### Diagnosis and management of thrombotic microangiopathy

At each of the participating centers, TMA was diagnosed by a multidisciplinary team including transplant specialists and nephrologists. The diagnostic criteria were evidence of significant microangiopathic hemolysis (schistocytes on peripheral blood smear, haptoglobin <0.15 g/L, and/or lactate dehydrogenase >1000 U/L)^26^ and thrombocytopenia (platelets <150·10^3^/mL or platelet-count decrease >25%) or kidney-biopsy findings of microvascular thrombosis with damage to arteriolar and capillary walls.^3^

Underlying causes or triggers of TMA were sought. ADAMTS 13 activity (%) at the time of TMA was measured. To detect innate or acquired thrombophilia, patients were tested for anti-phospholipid antibodies (lupus anticoagulant, anticardiolipin, and antibeta2 glycoprotein antibodies), C protein, S protein and anti-thrombin III deficiencies, resistance to activated protein C, and factor V Leiden and prothrombin gene mutations. HIV, cytomegalovirus (CMV), HCV, and parvovirus B19 viremia were quantified in blood and Shiga toxin in stool. When CNI toxicity was suspected, the management options included dosage reduction, switching to a different CNI, or discontinuation in favor of belatacept. This decision was guided by a multidisciplinary evaluation. Infection as a potential trigger was systematically investigated and managed as appropriate.

The additional treatment combined supportive care and targeted therapies. Among supportive measures, symptom-directed care encompassed red-blood-cell and/or platelet transfusions as needed for severe anemia and thrombocytopenia, dialysis for acute kidney injury refractory to conservative management, treatment of hypertension, and broad-spectrum antimicrobial therapy when infection was present. Optimization of the fluid and electrolyte balance was also part of this approach.

Plasmapheresis was used primarily in patients with clinical signs suggestive of thrombotic thrombocytopenic purpura (TTP) or complement-mediated TMA, notably in those with severe thrombocytopenia, microangiopathic hemolytic anemia, and neurological involvement. A typical course consisted of 5 to 7 sessions, adjusted according to the clinical and biological response.

Eculizumab was used selectively when complement activation was suspected or confirmed, based on evidence such as low serum C3/C4 levels and elevated soluble C5b-9. It was prescribed after a multidisciplinary-team discussion and administered for a median of 2.5 months (range: 1–6 months), depending on the patient's clinical course and response to therapy.

### Data collection

We used standardized forms to record demographic data, the medical history including the age at and reason for transplantation, the post-transplantation immunosuppressive regimen, and the features and outcome of TMA. Details on the indications and effects of CNI withdrawal and belatacept therapy were collected.

### Outcome measures

Survival after TMA was the primary outcome. The secondary outcomes were the clinical features of TMA, kidney function (estimated glomerular filtration rate, eGFR), development of acute lung-transplant rejection or CLAD, and management of TMA.

### Statistical analyses

The Shapiro-Wilk test was used to assess variable distribution. Continuous variables were described as mean±SD and compared by applying Student’s *t* test if normally distributed. Non-normal continuous variables were described as median [IQR] and compared using the Mann-Whitney U test. Categorical variables were described as n (%) and compared using the chi-square test or Fisher’s exact test, as appropriate.

The patients were divided into two groups based on whether they were kept on CNI therapy or switched to belatacept therapy. Time from transplantation to TMA and survival after TMA onset were evaluated using the Kaplan-Meier method and compared between groups using the log-rank test.

Cox regression was used to look for associations linking the baseline factors at transplantation and factors at TMA diagnosis, all listed in Table 4, to overall post-TMA survival. A multivariate model was built using the variables associated with *P* values below 0.1 by univariate analysis. Relative risks were computed with their 95% confidence intervals (95%CIs). Due to the large proportion of missing values (30%), analyses for factors associated with eGFR were not performed.

Two-tailed *P* values <0.05 were considered significant. All statistical analyses and graphs were performed using the R program v4.3.2 with the 'ggplot2', 'survival', 'cmprsk', and 'mice' packages (https://www.r-project.org).

## Results

### Patients and baseline characteristics

**Figure 1:**
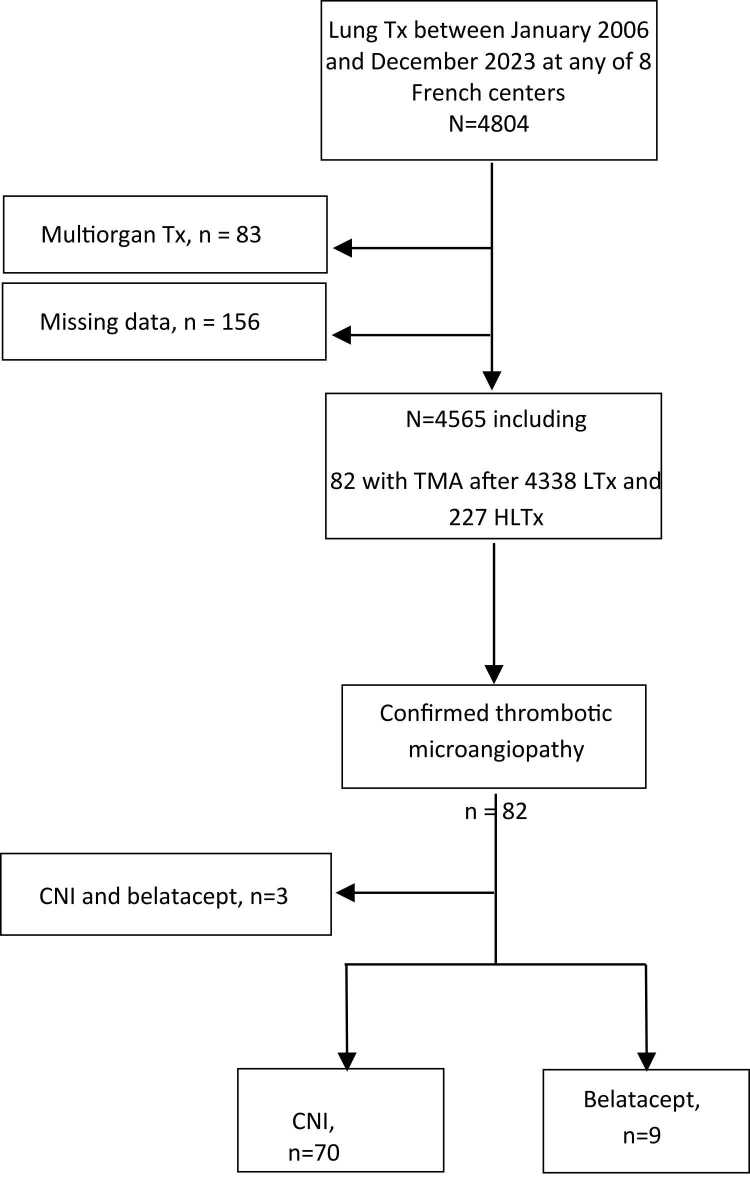
Flowchart of the study patients.

Figure 1 is the patient flowchart. The incidence of TMA was assessed in 4565 patients and the features and outcomes of TMA in 79 patients.

TMA developed in 82/4565 patients. The three patients in whom TMA was managed by continued CNI and added belatacept therapy were excluded. Table 1 reports the baseline characteristics of the 79 remaining patients, of whom 70 were kept on CNI therapy and nine were switched to belatacept therapy.

**Table 1:** Main Features of the 79 Lung or Heart–Lung Transplant Recipients Who Experienced Thrombotic Microangiopathy

	Overall n = 79	No Belatacept n= 70	Belatacept n = 9	*P* Value
Centers, n(%)				<0.01
Bordeaux	7 (9)	6 (9)	1 (11)	
Grenoble	4 (5)	3 (4)	1 (11)	
Le Plessis-Robinson	15 (19)	8 (11)	7 (78)	
Nantes	6 (8)	6 (9)	0	
Paris	17 (22)	17 (24)	0	
Suresnes	24 (30)	24 (34)	0	
Strasbourg	4 (5)	4 (6)	0	
Toulouse	2 (2)	2 (3)	0	
Male, n (%)	39 (49)	33 ( 47)	6 (67)	0.31
Recipient age, med [IQR]	50 [33 - 61]	50 [32 - 61]	57 [39 - 62]	0.32
Blood group, O/A/B/AB, n(%)	38 (48) /31 (39) /7 (9) /3 (4)	31 (44) / 30 (43) / 6 (9) /3 (4)	7 (78) / 1 (11) / 1 (11) / 0	0.16
CMV donor positive / recipient negative, n(%), n = 68	15 (19)	10 (14)	5 (56)	0.01
Transplantation indications, n(%)				0.02
Fibrosis	30 (38)	26 (37)	4 (45)	
Cystic fibrosis	19 (24)	18 (26)	1 (11)	
Chronic obstructive pulmonary disease	14 (18)	14 (20)	0 (0)	
Pulmonary arterial hypertension	6 (8)	3 (4)	3 (33)	
Other	10 (12)	9 (13)	1 (11)	
Lung transplantation procedure DLT/SLT/HLT, n (%)	68 (86) /9 (11) / 2 (3)	59 (84) /9 (13) / 2 (3)	9 (100) / 0 / 0	0.50
High-emergency transplant program, n (%)	14 (18)	10 (14)	4 (44)	0.05
Pre transplant ECMO, n(%), n = 75	4 (5)	4 (6)	0	0.99
Cardiopulmonary bypass, n (%), n = 76	31 (41)	27 (39)	4 (44)	0.99
Right ischemic time, min, med [IQR]	280 [246 – 382]	281 [243 – 410]	273 [255 – 305]	0.88
Left ischemic time, min, med [IQR]	342 [280 – 407]	334 [276 – 405]	372 [342 – 411]	0.30
Induction, n (%), n = 75	38 (51)	34 (49)	4 (44)	0.73
Dialysis during intensive care unit stay n (%), n = 75	8 (11)	5 (7)	3 (33)	0.05
Primary graft dysfunction score Grade 3 at 72 h, n (%), n = 76	13 (17)	9 (13)	4 (44)	0.04
Ventilation time during intensive care unit stay, med [IQR], n = 75	2 [1−8]	2 [1−8]	14 [5−38]	0.03
Haemothorax, n (%), n = 76	9 (12)	6 (9)	3 (33)	0.07
One-year post transplant mortality, med [IQR]	0 [0 - 0]	[0 - 0]	[0 - 0]	0.14

CNI, calcineurin inhibitor; DLTx, double-lung transplantation; SLTx, single-lung transplantation; HLTx, heart-lung transplantation; RRT: renal replacement therapy

The CNI and belatacept groups were comparable for most variables. However, there was a significant difference in the distribution of treatments between centers, with the vast majority of belatacept treatments given at the Plessis-Robinson center. In addition, the belatacept group had significantly larger proportions of patients who were cytomegalovirus (CMV) donor-positive/recipient-negative and who had pulmonary fibrosis or pulmonary hypertension as the reason for transplantation. In the belatacept group, the higher proportions of patients who were in the high-emergency transplantation program and who required renal replacement therapy during the intensive-care-unit stay indicate greater disease severity at transplantation (Table 1).

### Characteristics and management of thrombotic microangiopathy

As shown in Table 2, the main suspected TMA trigger was drug toxicity, in patients on CNI therapy alone, combined CNI and mTOR inhibitor therapy, and CNI therapy plus an infection. Among patients on CNI therapy, nearly half (47%) had above-target trough levels at the time of TMA diagnosis. Of these, 30% (13/39) were receiving treatment with azoles. Moreover, the combination of CNI and mTOR inhibitor treatment appeared associated with a particularly high risk, as it accounted for more than 30% of the TMA cases, representing over 70% of the patients treated with this combination.

**Table 2:** Characteristics and Management of Thrombotic Microangiopathy, n=79

	Overall n = 79	No Belatacept n= 70	Belatacept n = 9	*P* Value
Diagnostic criteria, n(%)				0.44
Biology only	55 (70)	50 (71)	5 (56)	
Pathology	24 (30)	20 (29)	4 (44)	
immunosuppressive regimen at TMA, n(%)				0.34
Tacrolimus + mycophenolate mofetil + steroids	31 (39)	28 (40)	3 (33)	
Tacrolimus + mTOR inhibitor + steroids	30 (38)	28 (40)	2 (22)	
Other	18 (23)	14 (20)	4 (44)	
Azithromycin at TMA, n(%), n = 75	36 (48)	31 (46)	5 (62)	0.47
Residual levels of calcineurin inhibitor at TMA, pg/mL, med [IQR]				
Cyclosporine, n = 4	292 [167 – 402]	292 [150 – 405]	185 [185]	0.99
Tacrolimus, n = 74	11.6 [7.8 – 16.6]	11.6 [7.6 – 15.8]	9.6 [5 – 22.2]	0.72
Calcineurin inhibitor residual levels above target levels	37 (47)	33 (47)	4 (44)	0.99
Azole treatment at TMA diagnosis, n(%)	23 (28)	21 (30)	2 (17)	0.49
Posttransplant thrombotic microangiopathy delay, m	19 [6 – 34]	20 [7 – 37]	4 [3 – 13]	0.05
TMA severity, med [IQR]				
Thrombocytes (nadir), G/L	68 [45 – 103]	68 [45 – 106]	80 [46 – 96]	0.89
Hemoglobin (nadir), g/dL	7.1 [6.5 – 8.2]	7.1 [6.5 – 8.2]	7.0 [6.4 – 8.0]	0.79
Intensive care units stay,	29 (37)	24 (34)	5 (56)	0.27
Dialysis, n(%), n = 51	15 (19)	12 (17)	3 (33)	0.36
TMA etiology, n(%)				0.82
Calcineurin inhibitor alone	39 (48)	33 (47)	6 (67)	
Calcineurin inhibitor + mTOR inhibitor	18 (23)	17 (24)	1 (11)	
Calcineurin inhibitor + infection	7 (9)	6 (9)	1 (11)	
Infection	6 (8)	6 (9)	0	
mTOR inhibitor alone	6 (8)	5 (7)	1 (11)	
Calcineurin inhibitor + mTOR inhibitor + infection	3 (4)	3 (4)	0	
TMA management, n(%)				0.01
CNI switch	32 (40)	32 (42)		
CNI reduction dose	26 (33)	26 (37)		
Plasmapheresis alone	12 (15)	12 (17)	1 (12)	
Eculizumab alone	6 (8)	5 (7)	0	
Plasmapheresis and eculizumab	9 (11)	5 (7)	4 (44)	

CNI: calcineurin inhibitor; Tx, transplantation; TMA, thrombotic microangiopathy; RRT: renal replacement therapy; mTOR inhibitor: mammalian target of rapamycin inhibitor

The management of TMA consisted of a change of CNI in 32 (40%) patients and a reduction of the CNI dosage in 26 (33%) patients. In nine patients, CNI therapy was stopped and belatacept given, for a mean of 22 months (range, 21 days to 72 months). In this group, the time from transplantation to TMA was shorter than in the group kept on CNI therapy (4 vs. 20 months, *P*=0.05). Moreover, plasmapheresis and eculizumab were used more often in this group, indicating a more severe presentation of TMA than in the group kept on CNI therapy (*P*=0.01).

### Outcomes of thrombotic microangiopathy

**Figure 2:**
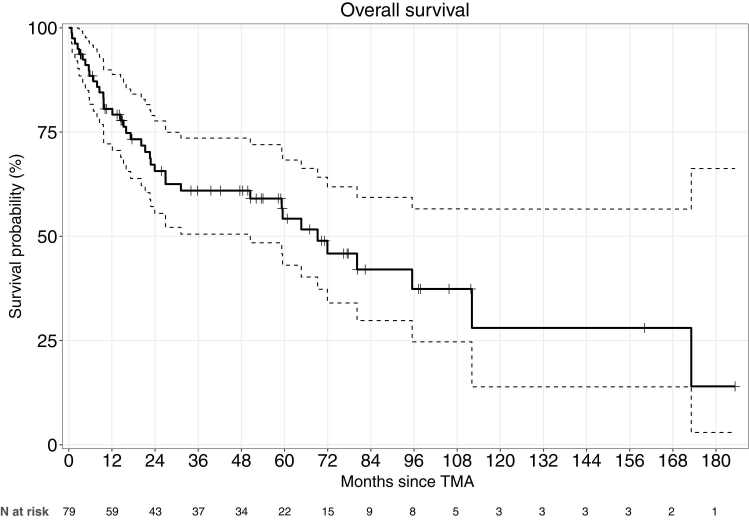
Kaplan-Meier estimates of survival after the diagnosis of thrombotic microangiopathy in the overall population of 79 patients. Survival rates were 80%, 61%, and 54% at 1, 3, and 5 years, respectively. TMA: thrombotic microangiopathy.

**Figure 3:**
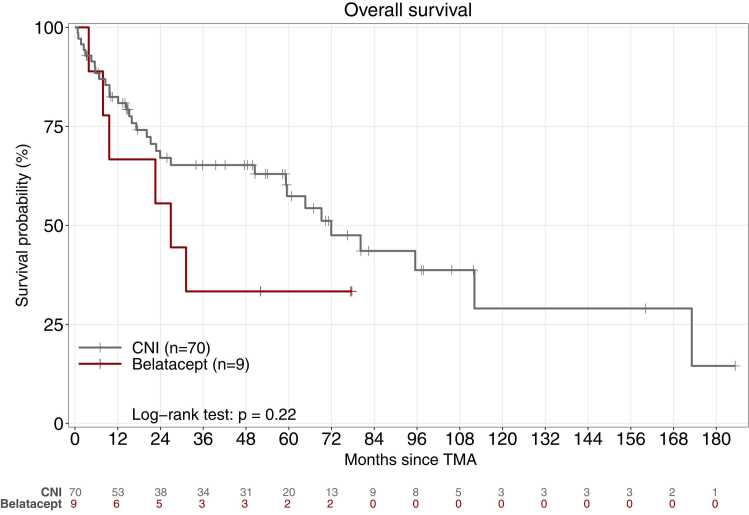
Kaplan-Meier estimates of survival after the diagnosis of thrombotic microangiopathy in the groups kept on calcineurin inhibitor therapy (n=70) and switched to belatacept therapy (n=9). Survival rates at 1, 3, and 5 years were 82%, 65% and 57% in the calcineurin-inhibitor group and 67%, 33%, and 33% in the belatacept group (*P*=0.22, log-rank test). TMA: thrombotic microangiopathy; CNI: calcineurin inhibitor.

Median follow-up after the TMA episode was 31 [11−66] months. At last follow-up, vital status was available for all 79 patients, of whom 38 had died (Table 3), 32/70 (45%) in the CNI group and 6/9 (67%) in the belatacept group. In the overall population, 1-, 3-, and 5-year post-TMA survival estimates were 81%, 61%, and 54%, respectively, with a median survival of 5.8 years (Figure 2). Survival rates 1, 3, and 5 years after the TMA episode were lower with belatacept, but the differences were not statistically significant (Figure 3; CNI: 82%, 65%, and 57%; belatacept: 67%, 33%, and 33%*; P*=0.22). The main causes of death were chronic lung allograft dysfunction (CLAD) [12/38 (32%)] and graft infections [12/38 (32%)]. Of note, there was a trend toward a higher frequency of graft-infection-related deaths in the belatacept group (42% vs. 11%, *P*=0.09) (Table 3).

**Table 3:** Outcomes in Patients Who Experienced Thrombotic Microangiopathy, n=79

	Overall n = 79	No Belatacept n= 70	Belatacept n = 9	*P* Value
Infection within the 2 post-thrombotic microangiopathy years, n(%)				
Bacterial pneumonia	0 [0 – 1]	0 [0 – 1]	1.5 [0.5 – 4]	0.01
Cytomegalovirus viremia	0 [0 – 1]	0 [0 – 0]	1 [0 – 1]	0.06
Aspergillosis invasive infection	0 [0 – 0]	0 [0 – 0]	0 [0 – 0.5]	0.04
Estimated glomerular filtration rate, mL/min/1.73 m^2^, med [IQR]				
Before thrombotic microangiopathy diagnosis	56 [38 – 90]	53 [37 – 89]	49 [45 – 86]	0.54
Nadir during thrombotic microangiopathy diagnosis	18 [13 – 30]	18 [13 – 31]	18 [10 – 25]	0.85
1-year after thrombotic microangiopathy	45 [33 – 72]	43 [33 – 62]	73 [8 – 129]	0.07
Acute cellular rejection within the 2 post thrombotic microangiopathy years, n(%), n = 74	13 (18)	11 (16)	2 (29)	0.69
Antibody-mediated rejection within the 2 post thrombotic microangiopathy years, n(%), n = 76	6 (8)	5 (7)	1 (8)	0.99
Chronic lung allograft dysfunction at last follow-up, n (%), n=81	24 (31)	21 (30)	3 (33)	0.99
Causes of deaths, n (%)				0.09
Chronic lung allograft dysfunction	12 (38)	12 (17)	0	
Pneumonia	10 (26)	7 (10)	3 (25)	
COVID−19	2 (5)	1 (1)	1 (17)	
Acute rejection	3 (8)	3 (4)	0	
Cancer	2 (5)	2 (3)	0	
Unknown	2 (5)	2 (3)	0	
Other	7 (18)	5 (7)	2 (17)	

CNI: calcineurin inhibitor; Tx, transplantation; TMA, thrombotic microangiopathy; RRT: renal replacement therapy; mTOR inhibitor: mammalian target of rapamycin inhibitor, COVID-19, coronavirus 19

Infections were more common in the belatacept group, usually with a significant difference (Table 3). Acute cellular rejection was non-significantly more common with belatacept. One year after the TMA episode, only 4 (7%) required durable renal replacement therapy without significant difference between the CNI and belatacept subgroups (*P*=0.42). However, a trend toward a higher eGFR was found in the belatacept group compared to the CNI group (Table 3; belatacept: 73 [8−129]; CNI: 43 [33−62]; *P*=0.07).

**Figure 4:**
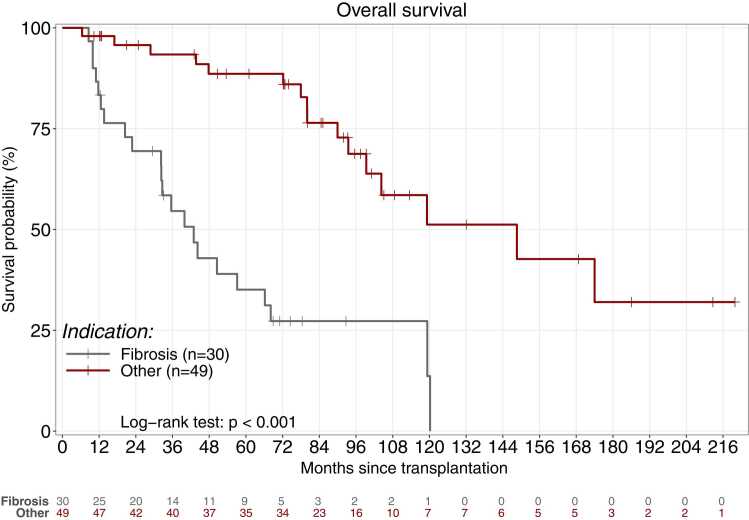
Kaplan-Meier estimates of survival without chronic lung allograft dysfunction (CLAD) after the diagnosis of thrombotic microangiopathy in the groups kept on calcineurin inhibitor therapy (n=70) and switched to belatacept therapy (n=9). CLAD-free survival rates at 1, 3, and 5 years were 77%, 58%, and 46% in the calcineurin-inhibitor group and 56%, 33%, and 33% in the belatacept group (*P*=0.46, log-rank test). TMA: thrombotic microangiopathy; CNI: calcineurin inhibitor.

**Figure 5:**
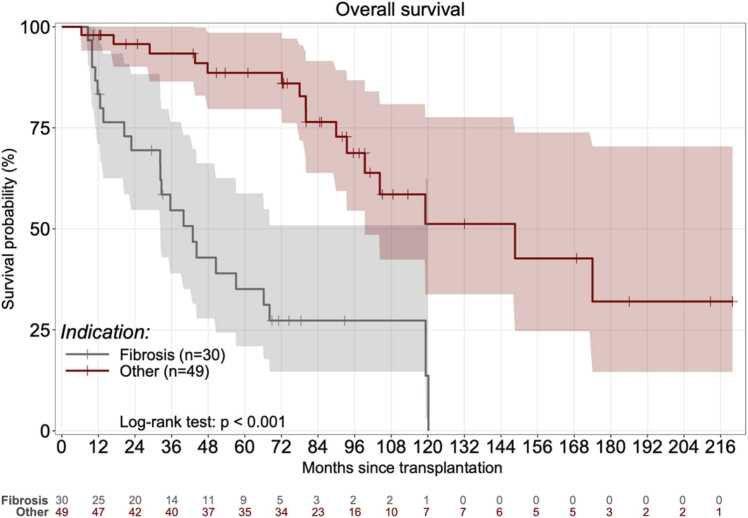
Kaplan-Meier estimates of survival after the diagnosis of thrombotic microangiopathy in the subgroups of patients transplanted for fibrosis (n=30) or for another indication (n=49). Survival rates at 1, 3, and 5 years were 83%, 55%, and 35% in group with fibrosis and 98%, 93%, and 89% in the group without fibrosis (*P*<0.01, log-rank test).

Table 4 reports the results of the univariate and multivariate analyses to identify variables associated with survival. Factors associated with worse survival by univariate analysis were older age at transplantation, transplantation for pulmonary fibrosis, and use of cardiopulmonary bypass. By multivariate analysis, only pulmonary fibrosis as the reason for transplantation was associated with lower survival. This result was confirmed by the Kaplan-Meier analysis (Figure 5). The subgroup analysis showed that the main difference was older age in patients with fibrosis compared to other transplantation indications (57 vs. 38 years, *P*<0.01), a possible factor of poorer post-transplant outcomes (data not shown).

**Table 4:** Univariate and Multivariate Analyses of Factors Associated with Post Thrombotic Microangiopathy Survival

	Reference	Modality	Univariate			Multivariate			
			HR	95%CI	*P* Value	HR		95%CI	*P* Value
Center	Suresnes	Bordeaux	1.53	0.33 – 7.18	0.59				
		Grenoble	0.01	0.01 – 99	0.98				
		Le Plessis Robinson	0.97	0.35 – 2.71	0.96				
		Nantes	2.21	0.67 – 7.24	0.19				
		Paris	3.39	0.36 – 8.45	0.33				
		Strasbourg	1.26	0.34 – 4.62	0.73				
		Toulouse	1.46	0.18 – 11.58	0.72				
Calcineurin inhibitor withdrawn + belatacept	No	Yes	1.72	0.71 – 4.15	0.23				
Age at lung transplant (years)	Continuous		1.03	1.00 – 1.05	0.04	1.03		0.99 – 1.07	0.12
Sex	Male	Female	0.64	0.33 – 1.21	0.17				
CMV D positive/R negative	No	Yes	1.43	0.68 – 2.99	0.34				
Transplantation indication	Chronic obstructive pulmonary disease	Fibrosis	5.75	1.66 – 19.91	0.01	6.92		1.50 – 31.90	0.01
		Cystic fibrosis	1.67	0.43 – 6.48	0.46	4.82		0.72 – 32.45	0.11
		Pulmonary hypertension	1.75	0.69 – 10.76	0.54	1.60		0.12 – 22.06	0.72
		Other	2.01	0.44 – 9.20	0.37	4.71		0.78 – 28.35	0.10
Blood group	Group A	AB	1.22	0.47 –5.22	0.79				
		B	1.28	0.40 –4.15	0.68				
		O	0.88	0.44 – 1.79	0.73				
Lung transplant procedure	Double-lung	Heart-lung	4.08	0.95– 17.44	0.06				
		Single-lung	1.26	0.51 – 3.10	0.61				
High-emergency transplant program	No	Yes	1.65	0.75 – 3.65	0.21				
Cardiopulmonary bypass	No	Yes	1.91	0.99 – 3.70	0.06	1.83		0.87 – 3.86	0.11
Right ischemic time (minute)	Continuous		1.00	1.00 – 1.00	0.87				
Left ischemic time (minute)	Continuous		1.00	1.00 – 1.00	0.89				
Induction	No	Yes	0.62	0.32 – 1.20	0.16				
Dialysis during intensive care unit stay	No	Yes	0.57	0.20 – 1.67	0.31				
Ventilation days during intensive care unit stay (d)	Continuous		1.00	0.99 – 1.02	0.77				
Primary grade III graft dysfunction within 72 h	No	Yes	1.35	0.61 – 3.00	0.46				
Haemothorax	No	Yes	1.54	0.66 – 3.60	0.32				
Thrombotic microangiopathy diagnosis	Biology only	Pathology	1.60	0.78 – 3.32	0.21				
Immunosuppressive regimen at diagnosis	Tacrolimus + MMF + steroids	Tacrolimus + mTORi + steroids	0.91	0.42 – 1.96	0.80				
		Other	0.99	0.43 – 2.24	0.97				
Azithromycin	No	Yes	0.97	0.35 – 1.98	0.88				
Residuals level of calcineurin inhibitor at TMA (pg/L) of cyclosporin	Continuous		1.00	0.99 – 1.01	0.58				
Residual level of calcineurin inhibitor at TMA (pg/L) of tacrolimus	Continuous		0.98	0.93 – 1.03	0.40				
Calcineurin inhibitor residual levels over target levels	No	Yes	0.93	0.49 – 1.76	0.83				
Azole treatment at TMA diagnosis	No	Yes	0.99	0.50 – 1.96	0.97				
Post-tranplant TAM delay (months)	Continuous		1.00	0.99 – 1.01	0.89				
Hemoglobin level at TMA diagnosis (g/DL)	Continuous		0.97	0.78 – 1.21	0.81				
Platelets level at TMA diagnosis (G/L)	Continuous		1.00	0.99 – 1.00	0.20				
Intensive care unit at TMA diagnosis	No	Yes	1.61	0.83 – 3.10	0.16				
Dialysis at TMA diagnosis	No	Yes	1.31	0.62 – 2.80	0.48				
TMA etiology	Calcineurin inhibitor	Calcineurin inhibitor + mTORi	0.43	0.16 – 1.14	0.09				
		Calcineurin inhibitor + mTORi + infection	1.21	0.28 – 5.20	0.81				
		Calcineurin inhibitor + infection	0.88	0.30 – 2.58	0.82				
		mTORi	0.84	0.29 – 2.48	0.76				
		Infection	0.00	0.00 – 100	0.99				
TMA management	Standard of care	Eculizumab alone	0.74	0.17 – 3.16	0.69				
		Plasmapheresis alone	0.92	0.38 – 2.27	0.86				
		Both	1.08	0.41 – 2.85	0.88				

TMA: thrombotic microangiopathy; DLTx: double-lung transplantation; SLTx: single-lung transplantation; HLTx: heart-lung transplantation; MMF: mycophenolate mofetil; mTOR inhibitor: mammalian target of rapamycin inhibitor

Of note, of the three patients who continued CNI therapy and also received belatacept but were not included in the analysis, all developed bacterial pneumonia and CMV viremia, two experienced acute cellular rejection, and none developed CLAD. Renal function one year after TMA was stable in two of the three patients, whereas the third required the initiation of chronic dialysis. After a mean follow-up of 41 months, two patients were alive and one had died from COVID-19 infection.

## Discussion

In this retrospective observational multicenter cohort study, 82 of 4565 patients who underwent LTx or HLTx experienced an episode of TMA at a median of 19 [6−34] months, yielding an incidence of 1.8%. Median follow-up after the TMA episode was 31 [11−66] months and median survival was 5.8 years. Nearly half the patients had above-target trough CNI concentrations at the time of TMA diagnosis. CNI therapy was continued in 85% (n=70) of patients and replaced by belatacept therapy in 11% (n=9). The eGFR at one year was higher in the belatacept group, although not significantly so, perhaps due to the very small sample size. The low statistical power in our study should also be borne in mind when considering the higher frequencies of death, bacterial pneumonia, CMV viremia, and graft-infection-related deaths in the belatacept group: that the differences were not statistically significant may be ascribable to the small sample size. By multivariate analysis, only pulmonary fibrosis as the reason for transplantation was independently associated with higher mortality.

### Incidence

The observed TMA incidence of 1.8% is consistent with the 1.4–3.8% range reported previously.^27^, ^28^, ^29^, ^30^

### Causes: treatments

In our study, the proportion of TMA cases attributed to immunosuppressive treatment toxicity was very high, in keeping with earlier studies of TMA after LTx.^28^, ^30^, ^31^, ^32^, ^33^, ^34^, ^35^, ^36^ In the recent study by Rosseels et al. of 29 patients with TMA, the development of TMA prompted a modification of the immunosuppressive therapy in 76% of cases.^30^

More than 80% of toxicities in our cohort were ascribed to CNI alone or in combination with mTOR inhibitor therapy and/or infection. Combined CNI and mTOR inhibitor treatment accounted for more than 30% of TMA cases, representing over 70% of patients treated with this combination. Hachem et al. reported a 7-fold higher risk of TMA with combined tacrolimus and sirolimus.^28^ Benefits of switching to a different CNI have been reported in several studies.^28^, ^30^, ^32^, ^37^ A switch was successfully performed in 40% of our patients. Finally, 30% of patients with CNI overdose were receiving concomitant azole treatment, which may have contributed to increase CNI levels. Concomitant azole use requires CNI dosage adjustment and tight monitoring.^38^ Uremic syndrome developed in two LTx recipients with high CNI levels and concomitant macrolide therapy.^35^ However, in our study, the use of azithromycin did not differ between the groups (Table 2), suggesting no major involvement in the risk of TMA.

### Other causes

Infection is another etiological factor for TMA.^30^ In our cohort, 8% of patients had an infection at the time of TMA diagnosis. Infection may contribute to TMA via complement activation and/or the release of pro-inflammatory cytokines. Infection-related endothelial injury may add to the toxic effects of immunosuppressants, highlighting the importance of closely monitoring transplant recipients for evidence of infection. Of note, only 5 patients had CMV infection identified at the time of TMA diagnosis. This contrasts with the recent report by Rosseels et al. of a markedly higher frequency of CMV infection (34%) among LTx recipients with TMA, suggesting a potential pathogenic role for CMV.^30^ Although, this discrepancy may appear surprising, differences in study design, timing of virological assessments, and thresholds for CMV detection may have led to under-recognition of subclinical or prior CMV reactivation in our population.

Other possible contributors to TMA after solid-organ transplantation are ischemia-reperfusion injury, coincidental disease, and relative ADAMTS13 deficiency.^39^ Time from transplantation to TMA onset is longer after LTx compared to liver and intestinal transplantation, suggesting differences in pathogenesis.^39^ Thus, findings from studies in recipients of other solid organs may not apply to lung recipients.^13^, ^40^, ^41^ Although higher mortality has been reported in transplant recipients with vs. without TMA^30^, in our cohort survival rates one and five years after TMA were 81% and 51%, respectively, compared to 85% and 59% in the overall population of LTx recipients transplanted after 2010 in the ISHLT registry.^42^

### Belatacept

Belatacept selectively inhibits T-cell activation via co-stimulation blockade, thereby providing effective immunosuppression without the direct nephrotoxic effects of CNIs.^43^, ^44^ Belatacept to prevent kidney rejection was approved in June 2011 in Europe and the US based on two Phase III randomized controlled trials, in recipients of kidneys from standard-criteria and extended-criteria donors, respectively, showing better kidney function with belatacept.^15^, ^16^ Other studies in stable kidney recipients also documented better kidney function with belatacept compared to CNI therapy.^17^, ^18^, ^44^ Conversion to belatacept in kidney recipients with CNI-induced nephrotoxicity improved renal outcomes.^18^, ^45^ Of note, in a randomized trial in stable kidney recipients who were either kept on CNI or switched to belatacept, although eGFR was higher with belatacept, none of the other outcomes, including adverse events, differed between the two groups.^17^ However, adverse outcomes have been recorded with belatacept including higher rates of acute rejection and post-transplantation lymphoproliferative disorder compared to CNI therapy.^16^, ^46^

Given that TMA usually affects the kidneys, conversion from CNI to belatacept may have therapeutic benefits regardless of the transplanted organ. Moreover, many LTx and HLTx recipients have renal dysfunction before or after transplantation. However, data on belatacept in recipients of non-kidney solid organs are scant. Two small retrospective studies in LTx recipients produced apparently conflicting results. In ten LTx recipients, including nine with severe renal failure, belatacept was associated with renal function improvements but also with a high frequency of severe acute rejection.^20^ Of note, a limited-intensity belatacept protocol was used in this study. The other study included 11 LTx recipients.^21^ Acute cellular rejection was not significantly more common after than before belatacept initiation, but the sample size provided very low statistical power. A small pilot randomized controlled trial in 27 LTx recipients compared belatacept to mycophenolate mofetil, with tacrolimus and prednisone in both arms.^47^ Three patients in the belatacept group died vs. none in the control arm, prompting premature trial discontinuation. Finally, in 37 lung recipients managed with belatacept and CNI-dose lowering, renal function improved but profound leukopenia and opportunistic infections resulted in substantial morbidity and mortality.^23^ In our study, belatacept was associated with a higher eGFR one year after the TMA episode. Infections were more frequent in the belatacept group, and there was also a trend toward a higher number of deaths, particularly due to infections. Moreover, although the risk of infection may have been increased by greater use of plasmapheresis and eculizumab in the belatacept group, these two treatments were not associated with survival in the Cox analysis, suggesting an independent role of belatacept in the increased risk of infection. That the differences in mortality were not significant should be viewed with circumspection given the very low statistical power. More specifically, the mortality rates of 46% in the CNI group and 67% in the belatacept group deserve attention. Consequently, we believe our findings do not support the use of belatacept as an alternative to CNI therapy in patients with TMA. However, this belief rests on the limited data available at present, and studies in larger cohorts are needed to address this issue.

### Limitations

Our study has major limitations. The design was retrospective and neither the diagnostic nor the therapeutic protocols were standardized across the participating centers. Second, the treatment of TMA has changed over time, particularly since the advent in the 2010s of eculizumab and belatacept. However, only a minority of patients (9 out of 79) developed TMA before 2010 and were therefore were unable to benefit from these medications. Third, the main limitation is the small sample size, notably the very small number of patients switched from CNI to belatacept therapy, which requires that non-significant differences be viewed with great caution.

## Conclusion

TMA after LTx and HLTx remains a rare but serious complication, predominantly associated with CNI toxicity and, to a lesser extent, infection. Our study underscores the critical need for stringent CNI monitoring protocols and prompt identification and management of infectious triggers to mitigate the TMA risk. In cases of suspected CNI-induced TMA, a cautious, stepwise approach—beginning with dose adjustment or switching within the CNI class—should be prioritized. Conversion to belatacept may be considered in selected cases, particularly when nephrotoxicity is pronounced and other measures have failed; however, clinicians should be acutely aware of the potential for increased infectious complications and currently unproven survival benefits in this population. Until robust prospective data are available, we advise that belatacept use remain exceptional and carefully individualized, within a multidisciplinary framework. Further prospective studies are needed to establish clear therapeutic algorithms and to assess the long-term risk/benefit profile of alternative immunosuppressive strategies in this context.

## Financial disclosure statement

None of the authors has any financial conflicts of interest to disclose regarding this study.

No funding was received for this study.

## Data-sharing statement

The data of this study will be shared with other investigators upon request to the corresponding author.

## Declaration of Competing Interest

The authors declare that they have no known competing financial interests or personal relationships that could have appeared to influence the work reported in this paper.
